# Small-scale on-site treatment of fecal matter: comparison of treatments for resource recovery and sanitization

**DOI:** 10.1007/s11356-021-12911-z

**Published:** 2021-03-05

**Authors:** Mariya E. Kelova, Aasim M. Ali, Susanne Eich-Greatorex, Peter Dörsch, Roland Kallenborn, Petter D. Jenssen

**Affiliations:** 1 Faculty of Environmental Sciences and Natural Resource Management (MINA), Norwegian University of Life Sciences (NMBU), Fougnerbakken 3, NO-1433 Ås, Norway; 2 Faculty of Chemistry, Biotechnology and Food Science (KBM), Norwegian University of Life Sciences (NMBU), Chr. M. Falsens vei 1, NO-1433 Ås, Norway; 3 Department of Contaminants and Biohazards, Institute of Marine Research, NO-5817 Bergen, Norway

**Keywords:** Feces, Dry toilet, On-site sanitation, Composting toilet, Resource recovery, Pharmaceuticals, Human excreta

## Abstract

On-site small-scale sanitation is common in rural areas and areas without infrastructure, but the treatment of the collected fecal matter can be inefficient and is seldom directed to resource recovery. The aim of this study was to compare low-technology solutions such as composting and lactic acid fermentation (LAF) followed by vermicomposting in terms of treatment efficiency, potential human and environmental risks, and stabilization of the material for reuse in agriculture. A specific and novel focus of the study was the fate of native pharmaceutical compounds in the fecal matter. Composting, with and without the addition of biochar, was monitored by temperature and CO_2_ production and compared with LAF. All treatments were run at three different ambient temperatures (7, 20, and 38°C) and followed by vermicomposting at room temperature. Materials resulting from composting and LAF were analyzed for fecal indicators, physicochemical characteristics, and residues of ten commonly used pharmaceuticals and compared to the initial substrate. Vermicomposting was used as secondary treatment and assessed by enumeration of *Escherichia coli*, worm density, and physicochemical characteristics. Composting at 38°C induced the highest microbial activity and resulted in better stability of the treated material, higher N content, lower numbers of fecal indicators, and less pharmaceutical compounds as compared to LAF. Even though analysis of pH after LAF suggested incomplete fermentation, *E. coli* cell numbers were significantly lower in all LAF treatments compared to composting at 7°C, and some of the anionic pharmaceutical compounds were detected in lower concentrations. The addition of approximately 5 vol % biochar to the composting did not yield significant differences in measured parameters. Vermicomposting further stabilized the material, and the treatments previously composted at 7°C and 20°C had the highest worm density. These results suggest that in small-scale decentralized sanitary facilities, the ambient temperatures can significantly influence the treatment and the options for safe reuse of the material.

**Supplementary Information:**

The online version contains supplementary material available at 10.1007/s11356-021-12911-z.

## Introduction

Only 38% of the global population have access to an improved sanitation facility connected to centralized treatment (WHO and UNICEF 2017). Hence, the majority of the remaining 72% uses on-site sanitation systems. Such systems are common in areas without or only with minor municipal infrastructure and in rural, remote, and small settlements and are commonly dry sanitation systems (WHO and UNICEF 2017). They have low resource input – no water and no complex and expensive infrastructure and, depending on the system, use little to no electricity (Tilley et al. 2014). The fecal sludge in those systems consists of mostly urine and feces and therefore is concentrated in a small, undiluted volume with high concentration of macro- and micronutrients, as well as organic matter, which can be valuable inputs to the surrounding agroecosystems. However, the fecal sludge is also associated with hazards as pathogens and micropollutants, including pharmaceuticals and other chemicals of emerging concern (Schönning et al. 2007; Hester and Harrison 2016; de Oliveira et al. 2020; Gros et al. 2020).

Most on-site sanitation systems do not treat the fecal sludge to facilitate safe reuse (WHO and UNICEF 2017). Currently, the common practices are not considering treatment or resource recovery and rely on subsequent storage or disposal (Strande and Brdjanovic 2014; Tilley et al. 2014). Dry composting toilets are considered one of the best current options for on-site treatment in terms of resource recovery (Orner and Mihelcic 2018; McConville et al. 2020). However, composting is not always successful, and the resulting material is usually neither stabilized nor sanitized (Niwagaba et al. 2009; Hill et al. 2013). Few studies have addressed this important aspect by examining how to improve the treatment physically or chemically by, i.e., solar heating (Redlinger et al. 2001), different bulking materials (McKinley et al. 2012; Hashemi et al. 2019), and/or amendments such as biochar (Hijikata et al. 2015) or urea (Vinnerås 2007). Others have focused on modifying the treatment by vermicomposting (Yadav et al. 2011), lactic acid fermentation (LAF) (Andreev et al. 2018), or fly larvae composting (Lalander et al. 2013a).

Combinations of treatments are considered promising. Integrated composting-vermicomposting has been investigated for a variety of organic wastes (Lim et al. 2016) including fecal slurry (Yadav et al. 2012). The material is first sanitized by thermophilic composting and conditioned further with earthworms to improve the quality of the end product. Pre-composting facilitates better conditioning because earthworms are vulnerable to thermophilic temperatures and toxic compounds in the organic wastes (Yadav et al. 2012). Another treatment combination for on-site sanitation is pre-treatment with LAF followed by thermophilic composting (Andreev et al. 2016) or vermicomposting (De Gisi et al. 2014). LAF is easy to manage and reduces quickly fecal pathogens, while the organic matter and nutrients are retained (Odey et al. 2018a). However, LAF alone does not sufficiently stabilize and sanitize fecal matter, and further treatment is needed before application as soil conditioner or fertilizer (Andreev et al. 2018). The combination of LAF and vermicomposting is part of the Terra Preta sanitation, which is inspired by ancient practices of organic waste management for soil fertility in the Amazon region (De Gisi et al. 2014). Central to the Terra Preta sanitation concept is the addition of carbonaceous pyrogenic material as biochar to retain nutrients and increase the product value for improving soil health and fertility. Biochar amendment in organic waste treatment has been shown to have benefits for agricultural application, with respect to retention of nutrients and pollution remediation (Wu et al. 2017). However, the efficiency for pollutant removal has not yet been assessed.

Biological transformations of fecal matter in on-site sanitation systems based on composting, vermicomposting, and LAF, even though considered as low-tech, can contribute to a cleaner local environment. If those practices are suitable in the local social and economic context, they have the potential to increase sustainability through recirculating nutrients and organic matter from excreta to agriculture and contribute to the currently propagated circular bioeconomy strategy. It is therefore important to explore different treatments in more detail and compare them directly with regard to the risks to human health and content of contaminants such as pharmaceutical residues and others, as well as assess their value for agricultural application.

Both composting and fermentation rely on biological processes, which are influenced by environmental conditions and management practices. Microbial transformations of the organic material are the foundation of these processes and are strongly influenced by, e.g., ambient temperatures. In small-scale systems, such as decentralized on-site sanitary facilities, the influence of temperature will be important. The fecal matter treatment in cold environments may be inhibited requiring different design considerations and management (Chen et al. 2020).

The aim of this study was to compare small-scale conventional composting with and without addition of biochar and to compare it to lactic acid fermentation (LAF) at three different ambient temperatures (7, 20, and 38°C). We further evaluated the use of vermicomposting at room temperature as a secondary treatment step. The composting process was investigated by measuring microbial activity and was compared to LAF by determining the changes in physicochemical characteristics and enumerating fecal indicators as well as quantifying selected pharmaceutical residues. After vermicomposting, the different treatments were evaluated with respect to changes in physicochemical characteristics, abundance of fecal indicators, and worm density.

## Materials and methods

In order to compare small-scale on-site sanitization strategies for fecal matter, a controlled laboratory experiment was carried out with three fecal matter mixtures, each run in three replicates at three different temperatures. All treatments were subjected to degradation (by composting or LAF) under controlled temperature for 71 days, followed by 15 days of composting and 77 days of vermicomposting at room temperature.

### Composting reactors and experimental setup

**Fig. 1:**
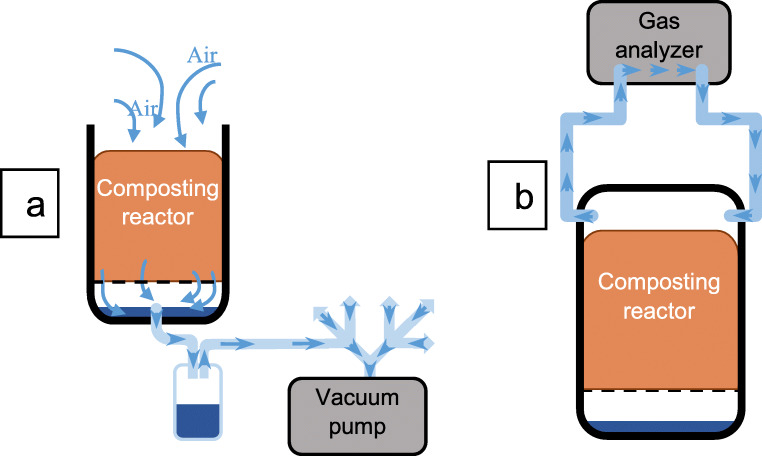
Schematic representation of the composting reactor setup. **a** When the pump is working. **b** When closed and connected to the CO_2_ analyzer

The experiment was conducted in tailor-made 16-L small-scale reactors. The reactor size was chosen, as a compromise between real-world size for on-site sanitation from a single toilet and the need for multiple replicates under controlled conditions. As suitable for the purpose, commercially available bokashi bins (0.38 × 0.33× 0.27 m) were used. The bins used for LAF were kept closed, whereas the reactors used for composting were modified by replacing the tap at the bottom with a tube connector. The connector was linked via 6-mm (inside diameter) tube to a liquid trap and a pump (Mini Diaphragm Vacuum Pump LABOPORT, model N86 KN.18, KNF, Freiburg, Germany) to pump air through the material (Fig. 1a). Further, two holes were drilled on the two top opposite short sides and connected with hoses (inside diameter 3 mm) to a gas analyzer (Fig. 1b). When the lid was closed, this created a closed circuit for the headspace air and allowed to determine rates of CO_2_ production. Aeration and CO_2_ measurements were operated sequentially.

**Fig. 2:**
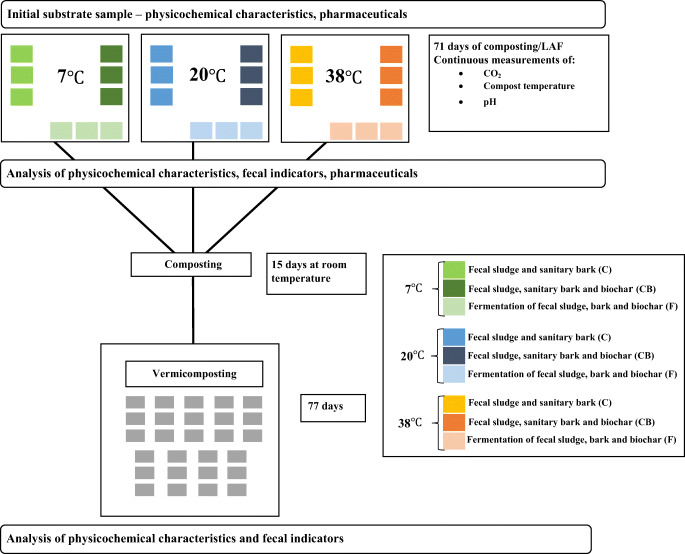
Overview of the experimental setup, treatments, and sampling timeline

Replicate reactors were placed in three climate-controlled rooms, maintaining ambient temperatures of 7, 20, and 38°C, respectively, for 71days. The temperatures were chosen to demonstrate a range of low, normal, and high ambient temperatures. The 7°C is representative of cold environments and 20°C of warm environments, and 38°C corresponds to additional heating, which increases the microbial activity and speeds up the composting (Sundberg et al. 2004; Eklind et al. 2007). At each temperature, there were three replicates of (1) composting mix from excreta with sanitary bark (Mix C); (2) composting mix with excreta, sanitary bark, and biochar (Mix CB); and (3) fermenting mix of excreta, sanitary bark, and biochar (Mix F) (Fig. 2). The compost reactors were dynamically incubated by sucking ambient air top-down through the substrate (negative aeration). For this, a vacuum pump was connected to the bottom of each of the six composting reactors (Fig. 1a) via equally long tubing to avoid pressure differences between the reactors. The pump was operated for 15 min followed by 30 min off. This aeration regime was chosen to avoid drying out of the substrate. The aeration regime was interrupted for measuring CO_2_ production and leachate pH. LAF reactors were incubated statically without aeration and closed lid. The moisture in the material was maintained by periodically returning the leachate collected in the liquid traps back into the composting mix and by sprinkling with tap water. After 71 days, the material from each reactor was emptied into another container, thoroughly hand-mixed with gardening tools, and subsampled for analyses.

Before vermicomposting, the reactors were mixed and sampled and left open for 2 weeks to compost at room temperature without forced aeration to increase pH and remove some of the NH_3_. Thereafter, 150 red wiggler worms (*Eisenia fetida)*, provided by industrial waste treatment and recycling facility Lindum, Drammen (Norway), were placed in each reactor. The reactors were kept moist and open and at room temperature (23°C). After 77 days, the material from each reactor was emptied in another container, the earthworms were counted and removed, and the material was thoroughly mixed with gardening tools and sampled. The method of counting did neither differentiate between development stages of the earthworms nor include eggs.

### Initial mixtures and materials

The initial substrates and corresponding amounts per reactor are listed in Table 1. The fecal material used for the experiment was mixed with small amounts of toilet paper and residues from sanitary pads and wet wipes. It was acquired from one collection compartment servicing five dry toilets at Åstjern cabin complex, Bleiken, Norway. The fecal sludge was accumulated over 2 years with daily fresh inputs until the day of collection. Separately collected urine was added to the mixture for mimicking fresh input to a dry toilet. The urine was obtained from a nearby farm household, collected, and stored in closed containers prior to use in this experiment. Bark was used as a bulking material to adjust the C/N ratio (based on preliminary tests with the materials) and to improve the structure. The bark was from commercially available packs of sanitary bark (Nordic Garden, Steinsholt, Norway) consisting of finely cut (0–15 mm) coniferous bark. It is commonly marketed and used as a dry toilet amendment in Scandinavia. Compost from a preliminary trial with fecal matter, bark, and food waste was used as inoculant. In the biochar treatments (Mix CB and Mix F), biochar – collected from a Pyreg pilot pyrolysis plant (established as part of the Stockholm Biochar Project (2017), and using garden waste as a substrate for the process) – was added to the substrate mix. The amount was corresponding to approximately 5% of the volume of the corresponding mixture. For the lactic acid fermentation treatment (Mix F), the substrate was inoculated with sauerkraut juice, instead of compost and biochar added, as in the Terra Preta sanitation concept (De Gisi et al. 2014). Sauerkraut is a widely available LAF product in Europe and had already been reported as inoculant for fermentation of feces (Andreev et al. 2016, 2017). The sauerkraut juice was drained from a mix from homemade and commercially available sauerkraut. Each substrate was combined and mixed in a cement mixer (Atika, model Comet 130 S, Ahlen, Germany) in two batches of 25–30 L and then distributed to the nine reactors for each treatment (Table 1). An initial substrate sample was taken from each reactor for chemical analysis.

**Table 1:** Initial substrate composition presented as average wet weight per reactor

	Fecal matter and sanitary bark	Fecal matter, sanitary bark, and biochar	Fecal matter, sanitary bark, and biochar, for fermentation
	(C)	(CB)	(F)
Fecal material (kg)	3.2	3	3.2
Sanitary bark (kg)	1.7	1.6	1.4
Compost inoculant (kg)	0.13	0.13	0
Urine (L)	0.75	0.75	0.7
Water (L)	0.5	0.47	0.25
Biochar (kg)	-	0.28	0.3
Lactic acid bacteria inoculant – sauerkraut juice (L)	-	-	0.3
Total (kg)	6.3	6.2	6.25

### Microbial activity in the composting treatments

The microbial activity during composting was followed by monitoring temperature continuously and by measuring respiration. Temperature was recorded with a HOBO Pendant Temperature Data Logger (Onset, Bourne, USA, 0.5°C accuracy). The loggers were buried in the center of the composting mix and remained there for the 71 days of treatment, logging at intervals of 10 min. Respiration was measured as CO_2_ accumulation in the reactor headspace using a portable CO_2_ infrared gas analyzer (EGM-5, PP-Systems, Amesbury, USA, dynamic range 0–100,000 ppm). Pump power and airflow rate were set to maximum, resulting in a circulating headspace airflow of ca. 0.5 L min^−1^. To measure respiration, the gas analyzer was sequentially connected to each reactor while keeping the lock closed (Fig. 1b). To determine the respiration rate, CO_2_ concentrations were recorded every 10 s for at least 6 min. The CO_2_ measurements were carried out daily during the first 15 days of composting and every second day for the remainder of the 71-day treatment period. Preliminary composting trials with a similar reactor, substrate volume, and substrate mixture showed that the highest activity occurred within the first 5 to 10 days.

The CO_2_ production rate was estimated from the increase of CO_2_ concentration over time by linear regression of on average 200 s from the middle of the 6-min measurement period and expressed as mg CO_2_-C reactor^−1^ day^−1^ using Eq. 1:

1 $$ {mgCO}_2-C\kern0.2em {reactor}^{-1}{hour}^{-1}=\frac{ppmC{O}_2{s}^{-1}\times {10}^{-6}\times V}{Vm\times M\times 3600\times 1000} $$

where ppm CO_2_ s^−1^ is the change in CO_2_ concentration, *V* is the volume of the headspace (*L*), *V*_m_ is the molar volume (L mol^−1^) at each temperature, and *M* is the molecular weight of C in CO_2_ (12 g mol^−1^).

Active degradation reduces the volume of the material with time. To account for the resulting increase in headspace volume, *V* was estimated based on the difference between the level of the material in the reactor and the lid at Day 1 and Day 71. The composting period was divided into 3 periods based on observations and confirmed by temperature and CO_2_ measurement: first, a period of intensive degradation – Day 1 to Day 5, then a period of active degradation – Day 6 to Day 20, and, finally, period of low activity – Days 21 to 71. The headspace volume was adjusted accordingly.

The total amount of C respired during composting was derived for each replicate by cumulating the average of each two adjacent measurements before averaging the values per treatment. The amount of cumulatively respired CO_2_-C was expressed per kg initial *C* in each reactor.

### Fecal indicators

The fecal indicators in the treatments were assessed by enumerating *Escherichia coli* and enterococci in composite samples taken in duplicates after the composting/fermentation and after the vermicomposting. The samples were stored at approximately 4°C and analyzed within 78 h of collection. A subsample of 10 g was diluted in 90-mL maximum recovery diluent (purchased from Sigma-Aldrich), and the mix was mechanically homogenized by a stomacher for 2 min. Preliminary samples from each treatment were used to determine the number of dilutions. They were further analyzed according to the method for the enumeration of *E. coli* by a defined substrate most probable number (MPN) technique (APHA 2005) using Colilert 18 test kits (IDEXX Laboratories Inc., Westbrook, ME, USA). The cell numbers were determined according to the IDEXX Quanti-Tray/2000 MPN table and expressed per g of dry solid.

### Physicochemical characteristics

Samples were collected from each container after 71 days of composting and after 77 days of vermicomposting. The material from each reactor was emptied into a larger container and thoroughly mixed with gardening tools before sampling. Dry matter and moisture content were determined by drying the samples at 60°C for 48 h. Volatile solids (VSs) were determined by combustion of dry samples at 500°C for 3–4 h in a muffle furnace. Total C was determined in crushed samples by dry combustion (Nelson and Sommers 1982) at 1050°C using a Leco CHN-1000 instrument (St. Joseph, Michigan, USA). Total N was measured on the same instrument according to the Dumas method (Bremner and Mulvaney 1982). Ammonium (NH_4_-N) was measured by flow injection analysis (FIA, Tecator FIAstar 5010 Analyzer, Hillerød, Denmark) after extraction with 2 M KCl in both fresh and dry samples. The difference in the concentration of NH_4_-N between fresh and dry samples was used to correct the tot N for the NH_4_-N loss as NH_3_ during drying. The pH was measured in leachate during the composting and at Day 77 of vermicomposting with a pH electrode (Orion™ ROSS Ultra, Thermo Fisher Scientific, Waltham, USA). pH was measured in solid samples with a wet sample to water volume ratio of 1:1.5. For the fermentation treatments, no leachate was collected because they were not subjected to aeration and did not require additional watering. Therefore, the pH was not monitored regularly but only measured twice during the first 10 days.

### Pharmaceuticals

For the quantification of targeted analytes in this study, a previously optimized analytical method was adopted with some modifications (Ali et al. 2019). The selection of the compounds (see Online Resource, Table S1) was based on their high rates of production and prescription in addition to their frequent detection in contaminated environmental samples in Norway.

Samples were prepared as described in Online Resource (S3) from initial mixtures and the products of composting/fermentation and analyzed with liquid chromatography-tandem mass spectrometry (see Online Resource, S4). The method performance characteristics are listed in Online Resource, Table S3 and described in S5.

### Statistics

Analysis of variance was used to compare the effects of the different treatments on the measured physicochemical characteristics. The assumptions were checked with Levene’s test for homogeneity of variances and the Shapiro-Wilk test for normality. Two-way ANOVA was used when the assumptions were met. To evaluate differences of means per factor, the ANOVA was followed by Tukey’s post hoc comparison of means (*p* < 0.5). The normality assumption was violated for concentrations of total N and NH_4_-N after vermicomposting, and the data were log transformed. For VS, NH_4_-N, and pH after composting and VS and total C after vermicomposting, the Kruskal-Wallis rank sum test was used for analysis. Differences between cumulative C-mass losses between the composting with and without biochar and initial and post-treatment concentrations of pharmaceutical compounds were evaluated with one-tail, unequal variance t-test. Statistical analyses were carried out using the R statistical package version 1.3.959 under the GNU public license (Boston, MA, USA).

## Results

### Effect of temperature on the composting process

**Fig. 3:**
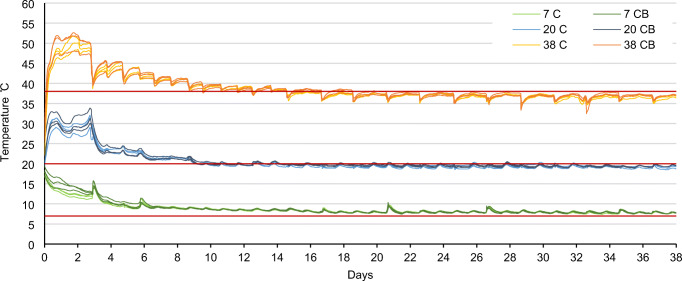
Temperature profile measured in the center of each reactor for the first 38 days of composting. The red lines represent the ambient temperature in the climate-controlled rooms – 7, 20, and 38°C. C composting, CB composting with addition of biochar

Microbial activity during the 71 days of composting was monitored as temperature change and CO_2_ production. Figure 3 shows the temperature profile for all treatments for the first 38 days of composting. The periodical fluctuations (positive in 7°C and negative in 38°C treatments) correspond to the time when the reactors were taken out of the climate-controlled room for weighing and pH measurements. In all treatments, the material was self-heated and maintained higher than ambient temperature during the first 6 to 10 days, but self-heating relative to ambient temperature was clearly larger at 38 than 7 and 20°C. The temperature profile shows clear differences between the treatments subjected to low, middle, and high ambient temperature; adding approx. 5% biochar to the mix had no effect on the released metabolic heat.

**Fig. 4:**
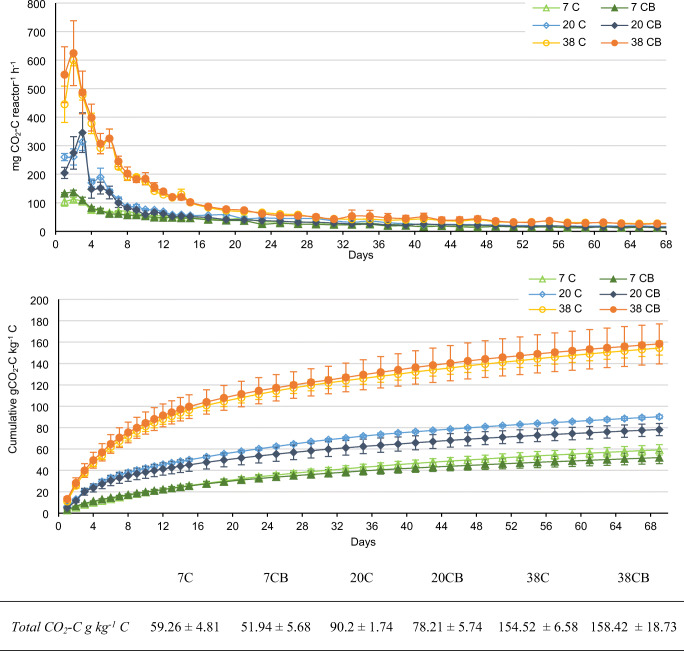
CO_2_ emission rates (top) and mean cumulative CO_2_ emission (bottom) (*n*=3, ± SD) during composting at three different ambient temperatures (7, 20, and 38°C). C composting, CB composting with addition of biochar. Cumulative CO_2_ emission estimation is based on periodic CO_2_ production measurements in the headspace and adjusted for initial total C. The average values with standard deviations for three replicates for total cumulative CO_2_-C g kg^−1^ C are given below in the table

The CO_2_ evolution rates for composting treatments are shown in Fig. 4 (top). Similarly to the temperature profile, highest CO_2_ production was detected during the first 5 days before levelling gradually off and stabilizing after 30–40 days of composting. At 38°C, microbial activity was highest, and the CO_2_ production rates remained above those of other temperature treatments throughout the entire period. Maximum observed respiration rates were 463–707 mg CO_2_-C reactor^−1^ h^−1^ on Day 2 for the 38°C treatment, 254–422 mg CO_2_-C reactor^−1^ h^−1^ on Day 3 for the 20°C treatment, and 100–146 mg CO_2_-C reactor^−1^ h^−1^ on Day 2 for the 7°C treatment. The highest ambient temperature resulted in the highest CO_2_ production. There was no indication that the addition of biochar affected rates or dynamics of CO_2_ production.

The cumulative CO_2_-C loss varied from 45.6 to 177.6 g C kg^−1^ C across treatments. Ambient temperature had a strong effect on the relative amount of respired C, whereas the addition of biochar lowered respiration losses only insignificantly (Fig. 4 bottom). Two to three times more C was respired at 38°C than at 7°C after 69 days. The 38°C treatment also had the steepest initial increase in cumulative respiration, emitting half of the totally respired C within 9 days, whereas 12 (without biochar) and 11 days (with biochar) were needed at 20°C and 18 and 16 days at 7°C.

### Physicochemical characteristics

The physicochemical characteristics in the initial mixtures suggested that the materials were well-suited as a feedstock for active composting (Table 2). The C/N ratios in the initial mixtures corresponded to a ratio that facilitates active degradation (Epstein 1997) with little recalcitrant carbon materials as indicated by the high content of VS. The moisture content was at the higher limit of what is considered optimal for composting, 40–70% (Guo et al. 2012). The pH was alkaline.

**Table 2:** Physicochemical characteristics for the initial mixtures and after 71 days of composting/fermentation. The data are means (*n*=3) and standard deviation. Capital “T” indicates means that are significantly different due to effect of temperature and capital “M” due to mixtures. *Note:* pH values measured in samples

		Moisture %	VS %	pH	Tot. C %	Tot. N %	C/N	NH_4_-N mg g^−1^
Initial	Mix C	68 ± 0.5	89 ± 0.3	8.4	48 ± 0.25	1.83 ± 0.00	26 ± 0.1	2.802
	Mix CB	68 ± 0.1	89 ± 0.4	8.5	49.5 ± 0.85	1.93 ± 0.04	26 ± 0.1	3.435
	Mix F	69 ± 1.5	89 ± 0.2	8.2	50.3 ± 0.57	1.87 ± 0.07	27 ± 1.3	4.61
After composting/fermentation	7 C	70 ± 1.8	88 ± 0.2	7.2	47.6 ± 0.24^M^	1.78 ± 0.15	27 ± 2.5	0.862 ± 0.031
	7 CB	72 ± 0.7	89 ± 0.3	7.2	49.6 ± 0.58	1.83 ± 0.02	27 ± 0.1	0.791 ± 0.050
	7 F	66 ± 0.4	89 ± 0.1	6.8	51.6 ± 0.58	1.83 ± 0.08^M^	28 ± 1.1^M^	3.905 ± 0.051
	20 C	71 ± 0.6	89 ± 2.6	7.1	47.6 ± 0.33^M^	1.76 ± 0.06	27 ± 1.1	0.101 ± 0.063
	20 CB	70 ± 0.6	86 ± 1.0	7.2	49.4 ± 0.60	1.82 ± 0.06	27 ± 0.7	0.093 ± 0.022
	20 F	67 ± 0.1	89 ± 0.5	6.8	49.9 ± 0.42	1.81 ± 0.03^M^	28 ± 0.7^M^	4.118 ± 0.292
	38 C	69 ± 0.4	85 ± 0.7	7.3	46.8 ± 0.10^M^	2.28 ± 0.05^T^	21 ± 0.5^T^	0.867 ± 0.145
	38 CB	69 ± 1.3	79 ± 3.6	7	49.7 ± 1.44	2.19 ± 0.04^T^	23 ± 1.1^T^	0.455 ± 0.166
	38 F	67 ± 0.8	89 ± 0.8	7.5	49.9 ± 0.78	1.7 ± 0.04^T,M^	29 ± 1.0^T,M^	4.236 ± 0.042
			K-W test	K-W test				K-W test
Temperature		ns	**.**	ns	ns	***	***	ns
Mix		***	***	ns	***	***	**	***
Temperature × mix		*	/	/	ns	***	***	/
Significance codes: ***0.001, **0.01, *0.05 , . 0.1 *ns* not significant								

**Fig. 5:**
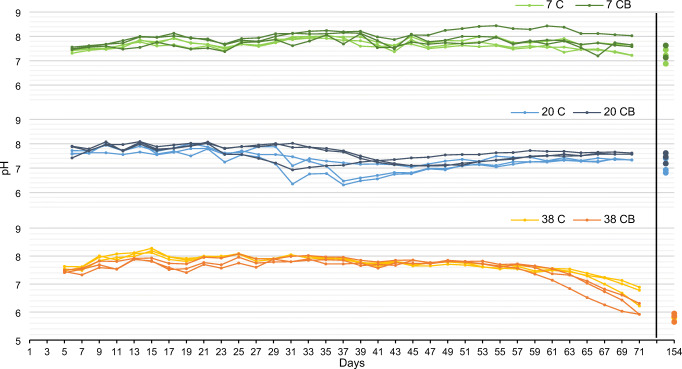
Leachate pH throughout 71 days of composting at 7, 20, and 38°C and on the final day of vermicomposting – Day 154. C composting material without biochar, CB composting with addition of biochar

The 71 days of treatment affected physicochemical properties differently in fermentation and composting treatments. The fermentation treatment resulted in a significantly higher C/N and NH_4_-N content, whereas no significant change was observed in the composting treatments. Clear differences between the treatments were observed in the 38°C composting treatments with lower C/N ratios, higher concentration of tot N, and lower VS. Furthermore, the pH in the leachate of 38 C and 38 CB decreased, which was not observed in the other treatments and indicates a chemical transition in the material that started around Day 57 (Fig. 5).

For the fermentation treatments, the pH was not followed continuously but was measured in leachate on Day 5 for 7 F as 4.5 and 20 F as 3.8 and on Day 9 as 4.3 for 7 F and 3.9 and 5.7 for two of the replicates in 20 F. The acidity in the leachate indicated successful inoculation with lactic acid bacteria and production of lactic acid in the first days. By contrast, the pH measured in the samples taken on day 71 indicated that the acidity was not maintained throughout the entire treatment period.

For the composting treatments, the changes of the pH in the leachate are plotted in Fig. 5. There was little change in the 7°C treatments. In the 20°C treatments, the pH decreased around Day 30 and returned back to neutral pH after about 20 days. This transient acidification was more pronounced in the compost without than with biochar. The pH in 38°C treatments was relatively stable throughout but started to decline during the last days of composting, i.e., after Day 57. The addition of biochar did not result in clear pH differences. However, in the 7°C and 20°C treatments, it resulted in slightly higher pH, whereas at 38°C, it resulted in lower pH values at the end.

### Fecal indicators

**Fig. 6:**
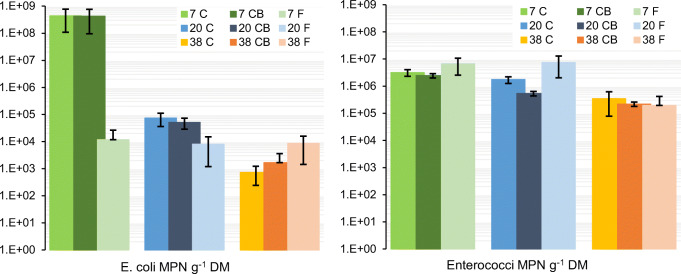
Enumeration of the indicator organisms *E. coli* (left) and enterococci (right) in samples after composting/fermentation at 7, 20, and 38°C ambient temperature. C composting, CB composting with addition of biochar. The error bars represent standard deviation

*E. coli* was detected in all treatments within the range of 90.6 MPN g^−1^ DM to the upper limit of detection >8.5 ×10^8^ (Fig. 6). The smallest MPN values of *E. coli* were detected in the 38 C treatment. *E. coli* was most abundant in 7 C and 7 CB, at the upper limit of detection and 4–6 log_10_ units higher than in the other treatments. Interestingly, at 7°C, the MPN *E. coli* in the fermentation was 5 log_10_ units lower than in the composting treatments.

Enterococci were detected in high numbers in all treatments (Fig. 6). The concentration varied with temperature with higher numbers at 7°C and 20°C and comparatively lower numbers at 38°C. The values for the 7°C and 20°C treatments were in the range of 6.4×10^4^–1.9×10^7^ MPN enterococci, whereas the treatments at 38°C had lower numbers in the range of 3.0×10^4^–1.3×10^6^.

For both *E. coli* and enterococci, the higher temperature treatments resulted in lower numbers. There was no clear effect of adding biochar on the abundance of fecal indicators. The fermentation treatments showed variable results depending on temperatures and had significantly lower numbers of *E. coli* after LAF at 7°C compared to composting at the same temperature.

### Fate of pharmaceutical compounds

The pharmaceutical compounds were selected based on their relatively high prescription rates in Norway, and their pattern and concentrations reflect the regional consumption and are subject to the variability within the matrix. The fecal matter and urine used in the study are not directly comparable with fresh excreta, and the initial concentrations were expected to primarily reflect compounds that partitioned to the solids, as those excreted with urine are more soluble and could have drained away. The initial concentrations were measured in the mixtures for direct comparison of concentrations before and after treatment.

The results for ibuprofen, sulfamethoxazole, and diclofenac must be regarded as semi-quantitative due to unreliable recoveries (see Online Resource, S5). The highest initial concentration among the detected pharmaceutical compounds was ibuprofen with a range of 8113–16551 μg kg^−1^. Ibuprofen was not detected in any of the composting products, but in the fermentation products, it was in the range of 93–212 μg kg^−1^. Sulfamethoxazole was detected in 7 out of 9 initial samples within the range of 3.5–11.7 μg kg^−1^ and after treatment only in 5 out of 27 samples in a range of 0.2–21.1 μg kg^−1^. Where detected in the products, amounts were lower than the initial values, with one exception where the concentration was 21.1 μg kg^−1^ (one of the 20 BC samples). Diclofenac showed interesting pattern and therefore is discussed alongside the other compounds.

**Fig. 7:**
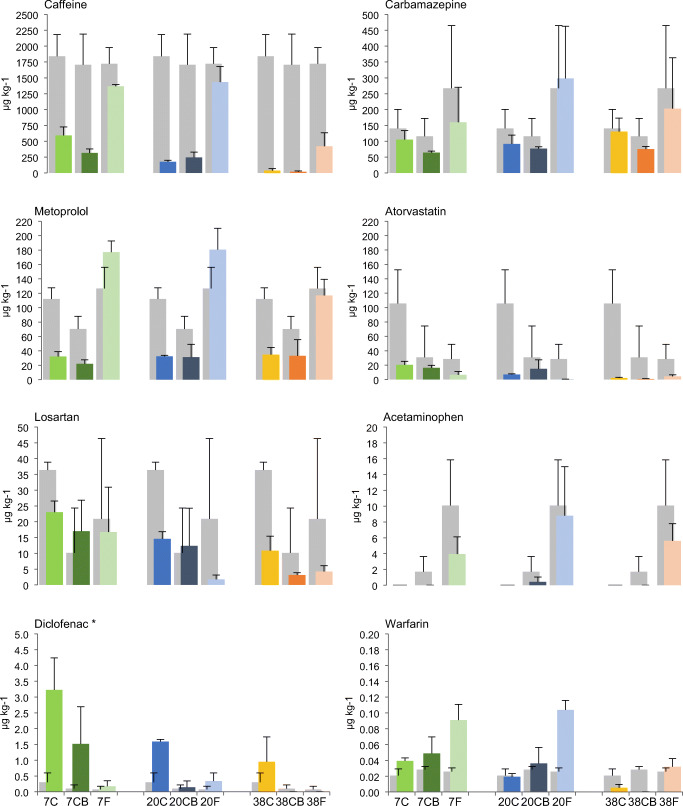
Comparison of caffeine, carbamazepine, metoprolol, atorvastatin, losartan, acetaminophen, diclofenac, and warfarin concentrations in the initial mixtures (gray columns) and after 71 days of composting/fermentation at 7, 20, and 38°C (colored columns). *The results for diclofenac are semi-quantitative. C composting, CB composting with addition of biochar. The error bars represent standard deviation

Figure 7 shows the detected concentrations for the other eight compounds, both in the initial and post-treatment samples. Caffeine was the compound with the second highest initial concentration with a range of 1351 to 2389 μg kg^−1^, whereas the concentrations of warfarin were the lowest with values of 0.012 to 0.034 μg kg^−1^ in initial samples. For caffeine, atorvastatin, losartan, diclofenac, and warfarin, the post-treatment concentrations were strongly negatively related to temperature, indicating that the increase in temperature and/or more active composting facilitated their removal.

Larger effects were observed when comparing the composting and LAF. For caffeine, carbamazepine, metoprolol, acetaminophen, and warfarin, there was a clear trend indicating more efficient removal during composting compared with fermentation. For caffeine, the results indicate higher removal in 38 C and 38 CB treatments and lower in the fermentation treatments. Carbamazepine showed low reduction in all treatments, and the highest concentrations were detected in the fermentation products. Post-treatment concentrations for metoprolol, acetaminophen, and warfarin showed a clear difference between composting and fermentation with larger reduction during composting. By contrast, atorvastatin, losartan, and diclofenac were detected in lower concentrations in the fermentation products compared to the composting products.

For most compounds, there was no clear effect of adding biochar, except for carbamazepine and diclofenac, for which the detected concentrations in the CB treatments were lower than those in C. For carbamazepine, the lowest detected concentrations were in the CB treatment.

**Fig. 8:**
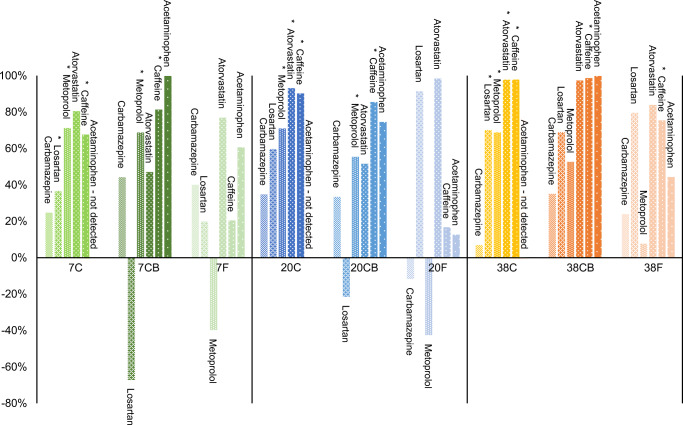
Removal of carbamazepine, losartan, metoprolol, atorvastatin, caffeine, and acetaminophen (in % of initial) after 71 days of composting/fermentation at 7, 20, and 38°C. The asterisk indicates the cases in which T-test showed statistically significant removal at *p*>0.05. C composting, CB composting with addition of biochar, F lactic acid fermentation

The removal within the different treatments is shown in Fig. 8 but should be interpreted with caution due to the high variation in the concentrations detected between the replicates. Statistically significant reduction in concentrations between initial and after treatment was found only in some treatments for caffeine, metoprolol, losartan, and atorvastatin (Fig. 8, with *). Diclofenac and warfarin are not plotted as they were detected in higher concentrations after treatment with some exceptions for the treatments at 38°C. Also, losartan was detected in 7 CB and 20 CB at higher average concentrations. Likewise, carbamazepine and metoprolol concentrations increased in the fermentation treatment products. This can be explained by cleaving back of conjugates or by change in efficiency of extraction due to changes in the chemical conditions and degradation of particles to which they may have been adsorbed to initially (Leclercq et al. 2009; Jewell et al. 2014).

### Vermicomposting

Vermicomposting further stabilized and conditioned the composing/fermentation products. The resulting material was visually similar to conventional vermicompost with no unpleasant odors. The stabilization was also evident from the significantly lower VS% and NH_4_-N content (Table 3) than before vermicomposting.

**Table 3:** Mean physicochemical characteristics (*n*=3, ±SD) for treatments after vermicomposting. The treatment factor refers to differences before and after vermicomposting. Capital “T” indicates means that are significantly different to the rest due to effect of temperature and capital “M” due to mixtures. *Note:* pH values measured in samples

	Moisture %	VS %	pH	Tot. C %	Tot. N %	C/N	NH_4_-N mg g^−1^
7 C	69 ± 0.6	87 ± 1.2	7.3	46.6 ± 0.51	1.98 ± 0.39	25 ± 3.2	0.026 ± 0.005
7 CB	71 ± 0.1	85 ± 0.9	7.4	48.7 ± 0.58	1.7 ± 0.01	29 ± 0.6	0.024 ± 0.004
7 F	70 ± 0.5	87 ± 0.6	7.8	48.3 ± 0.99	1.78 ± 0.07^M^	27 ± 1.5^M^	0.033 ± 0.002^M^
20 C	70 ± 0.6	87 ± 1.1	7.3	46.6 ± 0.33	1.67 ± 0.01	28 ± 0.1	0.02 ± 0.003^T^
20 CB	70 ± 0.2	87 ± 0.7	7.4	48.3 ± 0.40	1.69 ± 0.08	29 ± 1.3	0.02 ± 0.002^T^
20 F	70 ± 0.7	83 ± 2.0	7.5	48.5 ± 0.27	1.86 ± 0.10^M^	26 ± 1.3^M^	0.04 ± 0.007^T,M^
38 C	68 ± 1.0	86 ± 0.3	6.3	45.9 ± 0.50	2.19 ± 0.09^T^	21 ± 1.0^T^	0.018 ± 0.001
38 CB	68 ± 0.7	86 ± 0.5	6.4	47.9 ± 0.31	2.27 ± 0.08^T^	21 ± 0.9^T^	0.015 ± 0.003
38 F	70 ± 0.6	85 ± 1.0	7.4	49.9 ± 1.43	1.75 ± 0.02^T,M^	28 ± 1.1^T,M^	0.039 ± 0.008^M^
		K-W test		K-W test			
Treatment	ns	***	ns	**	**	***	***
Temperature	*	ns	*	ns	***	**	**
Mix	**	ns	ns	***	***	***	***
Temperature × mix	ns	/	**	/	***	***	**.**

Significance codes: ***0.001, **0.01, *0.05 , . 0.1; *ns* not significant

The physicochemical parameters after vermicomposting differed between the treatments, but followed similar patterns as those seen after composting/fermentation. The NH_4_-N concentration was still significantly higher in the previously fermented vermicompost than in the previously composted treatments, but on a lower level. Only the highest temperature treatment (38 C and 38 CB) sustained a high total N content and low pH, even after vermicomposting.

**Fig. 9:**
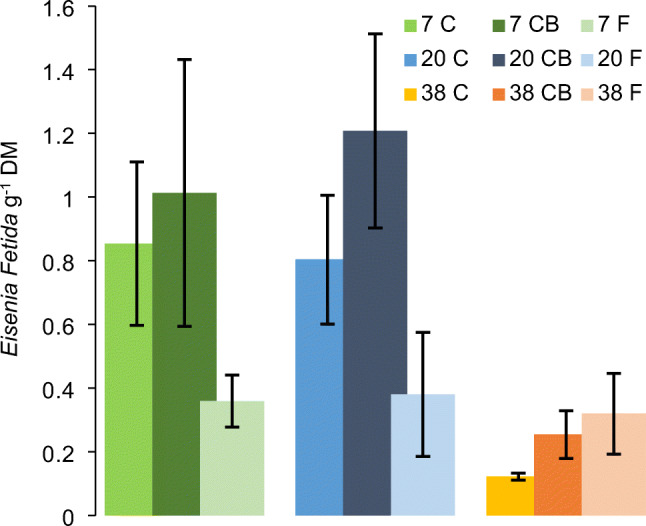
Mean density of *Eisenia fetida* (*n*=3, ± SD) after vermicomposting. The treatments were previously composted/fermented at 7, 20, and 38°C. C, composting, CB composting with addition of biochar

Figure 9 shows the density of *E. fetida* in the different treatments after 77 days of vermicomposting. The worms propagated in all treatments but varied in density within the range of 0.11–1.63 worms g^−1^ DM. Higher densities were detected in materials previously composted at 7°C and 20°C, whereas the lowest density is found in the 38°C material. In the CB treatments, there was a trend for higher average numbers of worms compared to the C treatments though not statistically significant.

**Fig. 10:**
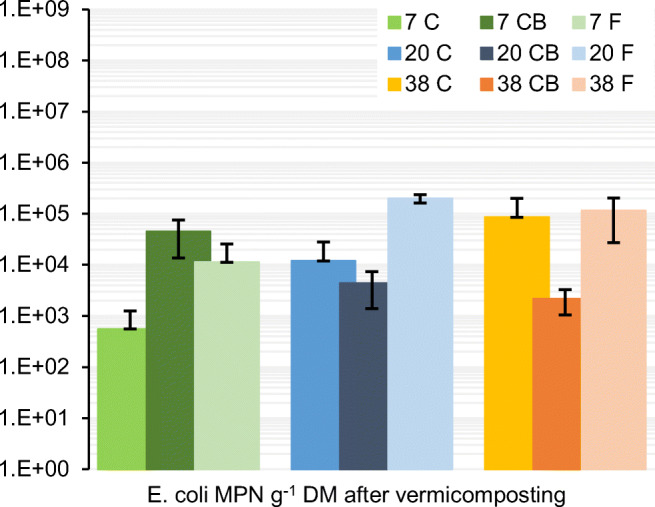
Enumeration of the indicator organisms *E. coli* after vermicomposting. The treatments were previously composted/fermented at 7, 20, and 38°C. C composting, CB composting with addition of biochar. The error bars represent standard deviation values

The *E. coli* cell numbers in the samples from the vermicomposting are plotted in Fig. 10. Compared to the composting/fermentation step, *E. coli* counts were reduced by 4–5 log_10_ during the vermicomposting for the 7 C and 7 CB treatments. However, in 20 C, 20 F, 38 C, and 38 F, higher cell numbers were detected after the vermicomposting indicating possible regrowth or contamination from the worms.

## Discussion

### Composting at different ambient temperatures

#### Microbial activity in the composting process

As evident from the temperature dynamics and the respiration rates, composting at 38°C supported a higher activity throughout the entire period, close to doubling the amount of respired C compared to composting at 20°C. The rate of the process depends on the availability of easily degradable substrates but also on maintaining optimal conditions such as temperature and aeration moisture (Haug 1993). It is likely that the higher temperature enhanced degradation by specific biota at a higher rate resulting in more available carbon for further degradation (Zhang et al. 2011; Wang et al. 2015), whereas at 7°C, the activity was low, and with time, the material became more compacted, the airflow was restricted, and accordingly the rate of degradation decreased. The lack of intensive degradation together with compaction, high moisture saturation, and no turning resulted in a sludge-like and water-logged material, which was not stabilized as indicated by the similar VS and C/N ratio in the substrate before and after the period of composting.

The temperature profile within the composting matrix is indicative of the process rate as a measure of the released heat but at the same time subject to the dynamics between heat production and heat loss to the environment. The conductivity of the material and the amount of composting substrate influence the temperature dynamics and can heighten temperatures or result in greater heat loss and affect the development of the process. Low ambient temperatures and lack of insulation result in higher net heat loss, which in turn slows microbial degradation (Niwagaba et al. 2009; Nasri et al. 2019). On the other hand, too high ambient temperatures can inhibit microbial activity as shown by Beck-Friis et al. (2001) for composting with external heating of 55°C.

As expected, the ambient temperature in these small-scale compost reactors without insulation had a significant effect on the composting dynamics, and an increase in the ambient temperature in the examined range between 7°C and 38°C resulted in more active and faster degradation and self-heating in the compost.

#### Physicochemical characteristics

The change in the physicochemical characteristics between the initial mixtures and the materials after treatment suggests that those exposed to 38°C had undergone most transformation. 38 C and 38 CB were characterized by lowest VS and C/N ratios and highest N contents, indicating the material was more degraded. In the 38°C composts, the increase in total nitrogen was likely due to a concentration effect caused by the weight loss associated with the mineralization of organic matter (Sánchez-Monedero et al. 2001; Guo et al. 2012).

In all composting treatments, the concentration of NH_4_-N was reduced; however, the changes in total nitrogen indicated that most of the NH_4_-N was immobilized or nitrified, rather than lost through ammonia volatilization. During composting, nitrogen transformations are affected by temperature, pH, feedstock, and aeration (Sánchez-Monedero et al. 2001; Sundh and Rönn 2002). Several factors may have contributed to the low N losses in this study: the relatively low temperatures of composting, suboptimal aeration levels, and returning the leachate back into the composting mix.

Measured pH dynamics indicated biochemical changes in the middle of the composting period for treatments at 20°C and at the end for those at 38°C and little to no change for those at 7°C. The commonly observed trend in pH during composting is an initial drop due to the formation of organic acids, which is then followed by an increase and stabilization at neutral pH (Epstein 1997). In this study, the quick initial drop due to organic acid formation could have happened before Day 5, before the first measurements of pH. An interesting phenomenon is the drop in leachate pH at the end of the composting at 38°C. One possible explanation is that it is a result of intensified nitrification, as the release of H^+^ in the nitrification process acidifies the composting mix. The study of Sánchez-Monedero et al. (2001) found a correlation between the concentration of nitrates and the pH. Supporting that explanation are also the higher values for total nitrogen with low percent ammonium in the treatments at 38°C.

#### Fecal indicators

Different ambient temperatures resulted in different post-composting numbers of indigenous *E. coli* and enterococci with higher numbers for the treatments at 7°C and lower for those at 38°C. In the treatments at 7°C, the MPN of *E. coli* was considerably higher than at the other temperatures and corresponded to what has been observed in fresh feces (Germer et al. 2010; Ogunyoku et al. 2016). They were therefore considered to represent the original levels of fecal indicators, to which the other treatments were compared to.

Inactivation of pathogens during composting is related to temperature and microbial activity in the compost and is due to heat inactivation and competition with the microflora promoted by the composting process (Haug 1993). Reduction of pathogens is commonly characterized by a time-temperature relationship and is measured by log_10_ reduction or a specified abundance limit of an indicator microorganism. Common references are sanitizing temperatures of 55°C for a few days (Schönning and Stenström 2004) or the limit of 1000 MPN *E. coli* g^−1^ DM in the final material (Jayathilake et al. 2019). In this study, sanitizing temperatures above 55°C were not reached in any of the treatments. Temperatures high enough to affect the survival of pathogens were recorded only for the reactors at 38°C. In the core of the composting mass in those treatments, the temperature exceeded 45°C in the first three days, and only in some, it reached temperatures above 50°C. However, even without sanitizing temperatures, the composting process resulted in 4×6 log_10_ lower cell numbers of *E. coli* in the more active composts at 20°C and 38°C in comparison to the treatments at 7°C and *E. coli* < 1000 MPN g^−1^ DM in treatment 38°C. Enterococci have a high survival rate in composts (Vinnerås 2007) and were less affected by the temperature during the composting.

During small-scale composting of fecal sludge, sanitizing temperatures are rarely achieved (Niwagaba et al. 2009; Hill et al. 2013), but the process can still be efficient for reducing the pathogenic load (Vinnerås 2007; Germer et al. 2010). The method by which the compost is applied can introduce further log reductions due to application to soil, fertilization of crops that are not to be consumed raw, or fertilization of food crops with eatable parts that are not in contact with the soil (World Health Organization (WHO) 2006; Schönning et al. 2007).

#### Fate of pharmaceutical compounds

Higher concentrations of pharmaceuticals can be expected in fecal sludge from source-separated sanitation compared to conventional wastewater (Butkovskyi et al. 2015; Gros et al. 2020). Ibuprofen and caffeine were the compounds detected at highest concentrations, which reflects their high consumption in Norway. Caffeine is in some cases considered a concern to the environment due to its high concentrations (Deblonde et al. 2011; Verlicchi and Zambello 2015). The initial concentrations of carbamazepine were also relatively high compared to what has been reported for wastewater and sludge (Martín et al. 2015; Verlicchi and Zambello 2015); nevertheless, Gros et al. (2020) reported higher concentrations in fecal sludge solids. Carbamazepine is a persistent, neutral compound that partitions to solids (Butkovskyi et al. 2015; Min et al. 2018; de Wilt et al. 2018), which can explain a possible accumulation over time in fecal solids.

The results of our study indicate a positive correlation between temperature and the removal/degradation of caffeine, atorvastatin, losartan, diclofenac, and warfarin. The concentrations in the treatment products were lower at a higher temperature. However, as shown, the composting was also more active at higher temperatures; thus, it is not possible to discuss the effects of temperature and composting activity separately. Both thermal decomposition and microbial transformation are possible mechanisms for the observed reduction. Most of those compounds have been shown to be biodegradable. Caffeine has been identified as an easily degradable compound (Deblonde et al. 2011; de Wilt et al. 2018). In comparison between mesophilic and thermophilic anaerobic digestion, Gros et al. (2020) demonstrated more efficient removal of atorvastatin in a thermophilic treatment.

#### Effect of the biochar

Addition of biochar had no clear effect on the measured endpoints in any of the temperature treatments. Addition of ~ vol. 5% biochar did not result in changes in the temperature profile or the dynamics of CO_2_ evolution. At 7°C and 20°C, though, the CB treatments had lower cumulative CO_2_ emissions and higher pH in comparison to the C treatments. There was no significant difference in the *E. coli* and enterococci between the compost with and without biochar. However, the pharmaceutical compounds carbamazepine and diclofenac had lower concentrations in the CB compared to the C treatment products. Carbamazepine is resistant to degradation and is mostly removed by sorption (Min et al. 2018; de Wilt et al. 2018), and biochar has been shown previously to be an efficient sorbent (Dalahmeh et al. 2018). Both carbamazepine and diclofenac are considered as high risk for the environment (Butkovskyi et al. 2016), suggesting that biochar addition in fecal matter subjected to composting can be used to mitigate the environmental effect of these compounds.

In a study on poultry litter composting, Steiner et al. (2010) found that 5% biochar had little to no effect, whereas addition of 20% biochar resulted in faster decomposition and lower nitrogen losses. Therefore, it would be interesting to investigate additions of biochar larger than 5%, particularly for its role for retaining nutrients and pharmaceuticals. However, larger amounts of biochar can result in alkaline pH, especially when composting organics with high initial pH, and thus inhibit composting through negative effects on the microorganisms (Khan et al. 2020). Different feedstocks, process conditions and amounts of biochar will give different results (Wu et al. 2017; Khan et al. 2020).

#### Lactic acid fermentation

Sufficient production of lactic acid and sustained acidity are key factors for the LAF process and elimination of pathogens (Odey et al. 2018b). In this study, lactic acid was not measured, and it was not possible to monitor the pH as little or no leachate was produced. The few pH measurements in leachate during the first days indicated acidification, but the pH measured in samples from Day 71 revealed that acidity was not maintained. It is therefore difficult to judge how successful was the LAF process.

#### LAF at different ambient temperatures

Comparison of physicochemical properties did not show significant effect of temperature on the LAF process. This was also indirectly confirmed by the results of the vermicomposting. Both the presence of fecal indicators and the density of worms after the vermicomposting did not differ significantly between LAF treatments conducted at different temperatures. There are no studies so far investigating LAF of fecal matter under different temperatures, but the existing literature suggests that higher temperatures (20–55°C) enhance the fermentation (Tang et al. 2016; Zhou et al. 2016).

#### Physicochemical characteristics

LAF products are typically characterized by low pH, high content of organic acids, and low decomposition (Andreev et al. 2017). The higher C/N ratio and high content of NH_4_-N in 7 F, 20 F, and 38 F corroborate this principal difference to the composting process. Studies have shown that LAF retains nutrients and organic carbon (Andreev et al. 2018). In the present study, even though post-treatment NH_4_-N was high, total nitrogen was similar to the composting treatments; therefore, this experiment does not confirm higher retention of nitrogen in LAF compared to composting. A probable explanation is a high retention of N in the composting treatments because the leachate was returned to the mix, as well as lower temperatures and therefore a lower activity in the compost as shown by the respiration data.

#### Fecal indicators

The MPNs of *E. coli* and enterococci suggest that the material was not properly sanitized (*E. coli* > 1000 MPN g^−1^ DM). Interestingly though, in 7 F, the *E. coli* numbers were approximately 5 log_10_ lower than in 7 C and 7 CB. LAF has been shown to efficiently reduce fecal indicator bacteria (Anderson et al. 2015; Andreev et al. 2017; Odey et al. 2018b). However, it has not been extensively researched whether LAF has a specific effect on fecal pathogens, nor whether the reduction in the indicator organisms is related to a reduction in other relevant pathogens like Salmonella, Ascaris sp., and viruses.

#### Fate of pharmaceutical compounds

To the best of our knowledge, there are no studies that have investigated LAF of fecal matter or wastewater in regard to its effect on pharmaceutical compounds. LAF is mostly utilized for food preservation and as such can be expected to have minimal effect on degradation of organic compounds. Our study confirmed degradation by LAF for most of the detected compounds, but post-treatment concentration of caffeine, ibuprofen, acetaminophen, metoprolol, and warfarin clearly suggested more efficient removal by composting than LAF. Caffeine and acetaminophen are easily degradable compounds (de Graaff et al. 2011; Deblonde et al. 2011; de Wilt et al. 2018). Ibuprofen has been shown to have high biodegradability in aerobic treatments and low in anaerobic treatments (de Graaff et al. 2011; Butkovskyi et al. 2016; Min et al. 2018; de Wilt et al. 2018). Metoprolol has been shown to be recalcitrant under anaerobic conditions and with better removal in aerobic compared to anaerobic conditions (de Graaff et al. 2011; de Wilt et al. 2018). The concentrations of carbamazepine varied between replicates but on average showed the same trend. In contrast, atorvastatin, losartan, and diclofenac were detected in lower concentrations in the fermentation products compared to the composting products. Those are anionic compounds with better sorption at low pH (atorvastatin pKa = 4.46, losartan pKa = 5.5, diclofenac pKa = 4.15 (PubChem 2020), Online Resource Table S1). Thus, removal of pharmaceutical compounds in LAF seems to be mostly due to sorption, whereas the main mechanism in composting seems to be aerobic biodegradation.

### Vermicomposting

Worms from the species *E. fetida* were introduced to the treatment mixtures after the first composting/fermentation step and multiplied in numbers in all treatments over a period of 77 days. The earthworms were successfully established in the 7°C and 20°C treatments but thrived less in the 38 C and all LAF treatments. Possible explanations could be the lower pH after 38°C, more stabilized organic material that has a lower food value for the worms, and the high NH_4_-N in the fermentation treatments (Edwards et al. 2010). It should be noted that the enumeration method did not differentiate between different development stages of *E. fetida* and therefore represents only a snapshot of the population in each treatment on the day of the collection. However, the comparison is comprehensive and supported by changes in physiochemical characteristics and *E. coli* MPN.

Vermicomposting further stabilized the material from all treatments, as evidenced by further reduction in VS and NH_4_ content. In that respect, the process significantly altered the 7 C and 7 CB treatments. The treatments at 7°C underwent less active composting, and the substrate was sludge-like after the first composting step. Earthworm activity during vermicomposting resulted in improved mixing and aeration and facilitated further decomposition and stabilization.

The composting treatments 7 C, 7 CB, and 20 C, 20 CB, which had the highest density of *E. fetida*, had the lowest numbers of *E. coli*, whereas the treatments with lower densities showed higher *E. coli* counts in comparison to after the composting. This negative relationship between worm density and *E. coli* counts suggests that vermicomposting as post-treatment of fecal matter can reduce the load of fecal pathogens, particularly after ineffective composting. An increase in the fecal indicators after vermicomposting could be due to regrowth of bacteria or contamination from the worms (Lalander et al. 2013b). Vermicomposting has been shown to reduce pathogens from dry sanitation systems (Hill and Baldwin 2012; Yadav et al. 2012; Lalander et al. 2013b). However, the studies so far have focused on indicator organisms, and there are knowledge gaps with regard to effects on variety of pathogens and correlations to vermicomposting parameters such as feedstock, worm density, and temperature.

### Practical significance

This study showed that ambient temperature has a significant effect on compost quality and removal of pathogens during on-site small-scale composting of fecal matter. In colder environments, this should be considered, as low temperature inhibits biological processes. Different options can be considered to ensure higher temperatures, such as heat preserving insulation (Vinnerås et al. 2003), addition of easily degradable substrate to trigger fast decomposition, and self-heating (Germer et al. 2010) or external heating. Insulation is an easy optimization, but it depends on a well-maintained composting process and self-heating of the substrate. Easily degradable substrates can come from domestic food waste but are subject to availability and of variable composition. External heating can be energy demanding, and in areas with high solar irradiance, passive solar heating could be a sustainable way to achieve higher temperatures (Redlinger et al. 2001; Kelova 2015).

Maintaining active composting can be limited by the local context due to environmental, economic, or cultural constraints. Composting activity is sensitive to moisture content, aeration, and bulking materials, and the control of these variables requires some level of expertise. Therefore, depending on the context, small-scale composting might not be the most suitable solution for on-site management of fecal sludge. By contrast, LAF does not require maintenance, can be operated in a shorter time period, and results in a reduction of *E. coli* comparable to composting at 20°C. It has therefore been considered a suitable option in an emergency context (Anderson et al. 2015). However, its product requires further treatment before application to agriculture. Vermicomposting is another option. In our study, the activity of the earthworms transformed and stabilized the material where composting was ineffective. Hill and Baldwin (2012) reported that vermicomposting toilets can produce more stable material with fewer fecal indicators in comparison to inefficiently managed composting toilets, the majority of which were operated at low ambient temperatures.

Recirculating the nutrients and organic matter from fecal matter back to the soil for crop and food production requires better understanding of the fate of pharmaceutical compounds that are released to the environment. We found that higher microbial activity and temperature in the compost resulted in more efficient removal of most of the investigated compounds. The main mechanism of removal of pharmaceuticals, therefore, is probably biodegradation under aerobic conditions. By contrast, LAF had minimal effect on the concentrations of most of the investigated pharmaceuticals, except for atorvastatin, losartan, and diclofenac, and sorption is assumed as a removal mechanism. Slowly degrading compounds such as carbamazepine, diclofenac, metoprolol, and losartan can still pose a risk to the environment; however, that can be mitigated by further treatment. Overall, combinations of LAF and composting for removal of pharmaceuticals would be an interesting enquiry due to the different effects these treatments have in combination, which might result in overall larger reduction.

## Conclusions

Our investigation compared composting of fecal matter with lactic acid fermentation under three different temperatures. Ambient temperature had a significant effect on the composting process and the quality of the resulting material. At 7°C, composting was less active, which resulted in limited transformation and material with high numbers of fecal indicators and pharmaceuticals. At 20°C, composting was more active, and the outcome was a more stabilized material with lower numbers of fecal indicators and more efficient reduction in concentrations of a variety of pharmaceutical compounds. At 38°C, the composting process resulted in the most stabilized and sanitized material. The addition of ~ vol. 5% biochar to the composting did not yield significant differences in the measured parameters. While the active composting at 20°C and 38°C yielded more stabilized material with less *E. coli* and pharmaceuticals, lactic acid fermentation was comparatively successful in reducing the number of *E. coli* at 7°C. The lactic acid fermentation, however, was not assessed with respect to lactic acid production and retained acidity, which limited the comparison with composting. The secondary treatment with vermicomposting resulted in further maturation and stabilization of the material in all treatments, and it was particularly beneficial in reducing *E. coli* numbers and transforming the substrates for the treatments that were previously composted at lower temperatures, i.e., 7°C and 20°C.

The results of our investigation highlight the limitations of composting at low temperature and how other treatments as lactic acid fermentation or vermicomposting can be a valuable alternative, particularly when composting is not successful. Therefore, depending on the local conditions, possibilities, and desired qualities of the end product, different alternatives for resource recovery can be considered. Sustainable utilization of the resources from on-site sanitation treatment of human excreta will also depend on expanding the knowledge on the nutrient values in these treatment products and how they can be best utilized in the local agroecosystems.

## Supplementary Information

ESM 1 (DOCX 141 kb)

## Data Availability

The datasets used and analyzed during the current study are stored in the institutional repository at the Norwegian University of Life Sciences (NMBU), Faculty of Environmental Sciences and Natural Resource Management and so are not publicly available. Data are however available from the corresponding author on reasonable request.
