# Supplementary material for: Small-scale on-site treatment of fecal matter: comparison of treatments for resource recovery and sanitization
Source: Environ Sci Pollut Res Int. 2021 Mar 5;28(45):63945–64. doi: 10.1007/s11356-021-12911-z (PMC8610962; doi:10.1007/s11356-021-12911-z)
Supplement: Supplementary file 1 — (DOCX 141 kb) [file 11356_2021_12911_MOESM1_ESM.docx]

**Small scale on-site treatment of fecal matter: comparison of treatments for resource recovery and sanitization**

Mariya E. Kelova^1*^, Aasim M. Ali^2,3^, Susanne Eich-Greatorex^1^, Peter Dörsch^1^, Roland Kallenborn^2^, Petter D. Jenssen^1^

**ENVIRONMENTAL SCIENCE AND POLLUTION RESEARCH**

^1^ Faculty of Environmental sciences and Natural Resource Management (MINA), Norwegian University of Life Sciences (NMBU), Fougnerbakken 3, Ås NO-1433, Norway

^2^ Faculty of Chemistry, Biotechnology and Food Sciences (KBM), Norwegian University of Life Sciences (NMBU), Chr. M. Falsens vei 1, Ås NO-1433, Norway

^3^ Current address – Department of Contaminants and Biohazards, Institute of Marine Research, Bergen NO-5817, Norway

* Corresponding Author

Mariya Kelova, email: [mariya.kelova@nmbu.no](mailto:mariya.kelova@nmbu.no)

# **Electronic Supplementary Material**

# **Analysis of Pharmaceuticals: Method description**

**S1. Chemicals and solvents**

The acetonitrile (CH_3_CN) and methanol (MeOH) used in solid phase extraction (SPE) and during chromatography were HPLC grade purchased from VWR (West Chester, PA, USA). Reagent grade CAN formic acid (CH_2_O_2_), and ammonia solution (NH_4_OH) were purchased from Sigma-Aldrich. The water used was Grade 1 purified with a Milli-Q water purification system (Millipore, Bedford, MA, USA).

**S2. Selection of Analytes**

The selection of the compounds in this study (see Table S1) was based on their high rates of production and prescription in addition to their frequent detection in contaminated environmental samples in Norway.

**S3. Sample Preparation**

In brief, an aliquot of 5.0 g sample from the initial mixtures and the products of composting/fermentation (wet weight, ww) was spiked with 10 µL of a mixture of internal standards (ISTDs) of 10 µg mL^-1^ concentration followed by 10 mL of extraction solution A (MeOH: CH_3_CN: H_2_O with 0.2% formic acid; 40:30:30). Subsequently, the mixture was mechanically shaken for 5 min and placed in an ultrasonic bath for 10 min and then centrifuged for 5 min at 3000 rpm using IKA Vibrax VXR vibrator (Janke & Kunkel, Staufen, Germany). After transferring the supernatant, the extraction was repeated using solution B (MeOH: CH_3_CN: H_2_O 0.1% NaEDTA and 0.2% NH_4_OH; 40: 30:30). The combined supernatants were diluted with 30 mL of Milli-Q water and directly concentrated by 500 mg Oasis HLB cartridges (Waters, Milford, MA, USA) which were conditioned with 6 mL of acetonitrile, followed by 6 mL of Milli-Q water. The cartridges were washed with 3 mL of 5% MeOH in water and dried under vacuum. Analytes were eluted with 6 mL volumes of MeOH into a glass tube and dried under a gentle stream of nitrogen at 37°C using a Reacti-Therm III evaporating unit (Thermo Fisher Scientific Inc., Rockford, USA). Ten microliters of DEET-d10 (10 µg mL^-1^) were added as a recovery standard and after adding 990 µL of 20 % CH_3_CN in water, the sample was then vortexed and subsequently filtered through a 0.2 μm microcentrifuge filter (Spin-X, Costar, Corning Inc., Corning, NY, USA). The resulting sample was transferred to polypropylene vials for immediate quantitative LC–MS/MS analysis.

## **S4. Liquid Chromatography-Tandem Mass Spectrometry (LC-MS/MS)**

## The analysis was conducted using an Agilent 1200 series HPLC (Agilent Technologies, Waldbronn, Germany) coupled to an Agilent 6490 (Agilent Technologies, Santa Clara, CA, USA) triple quadrupole mass spectrometer with an Agilent Jet Stream electrospray ion source (ESI). The column used for chromatographic separations was a Zorbax Eclipse plus C18 RRHD (2.1 x 100 mm, 1.8 μm) (Agilent, Palo Alto, USA) with a respective Guard Cartridge (4 μm x 3.0 mm ID) (Zorbax, Agilent, Palo Alto, USA). The mobile phase flow rate was 300 µL/min, the column oven temperature was 35°C, and the injection volume was 10 µL. The chromatographic separation was performed using binary gradient mobile phases, consisting of water with 0.1% formic acid (A) and 100% CH_3_CN (B). The initial mobile phase composition was 100% (A). B was linearly increased to 100% in 8.0 min and held for 7 min. Initial mobile phase conditions were restored over 1.0 min, and the column was allowed to equilibrate for 3 min, a total run time of 20 min. The parameters of ESI were as follows: gas temperature of 200°C, gas flow of 14Lmin^-1^, nebulizer 20 psi, sheath gas heater 250°C, sheath gas flow 11 10L min^-1^, and capillary voltage 3000 V. The ions were monitored in multiple reaction monitoring (MRM) and are listed in Table S2. Agilent MassHunter software (Version B.07.00 /Build 7.0.457.0, 2008) was used for instrument control, method validation and quantification.

**Table S1 Summary of the selected analytes with their molecular formula, structures, CAS numbers, supplier, and some physiochemical properties^[[1]](#footnote-1)^.**

| **Compound (Abbreviation)** | **Description** | **Mol. formula** | **Structure^[[2]](#footnote-2)^** | - **CAS Number** | **Supplier** | [**LogP**](http://www.acdlabs.com/logp)**^*^** | [**LogD**](http://www.acdlabs.com/logd) **(pH 7.4)^*^** | **LogK_OC_ (pH 7.4)^*^** |
| --- | --- | --- | --- | --- | --- | --- | --- | --- |
| Acetaminophen (ACE) | nonsteroidal anti-inflammatory | C_8_H_9_NO_2_ |  | - 103-90-2 | Sigma Aldrich, Oslo,  Norway | 0.34 | 0.40 | 1.6 |
| Caffeine (CAF) | - Psychostimulants | - C_8_H_10_N_4_O_2_ |  | - 58-08-2 | Sigma Aldrich, Oslo,  Norway | -0.13 | 0.28 | 1.5 |
| Carbamazepine (CAR) | - anticonvulsant | - C_15_H_12_N_2_O |  | 298-46-4 | Sigma Aldrich, Oslo,  Norway | 2.67 | 2.28 | 2.6 |
| Diclofenac sodium salt (DCF) | - nonsteroidal anti-inflammatory | - C_14_H_10_Cl_2_NNaO_2_ |  | 15307-79-6 | Sigma Aldrich, Oslo,  Norway | 4.06 | 1.37 | 5.0 |
| Ibuprofen (IBP) | - nonsteroidal anti-inflammatory | - C_13_H_18_O_2_ |  | 15687-27-1 | Sigma Aldrich, Oslo,  Norway | 3.75 | 0.45 | 0.29 |
| Metoprolol (MTP) | - β-blocker | - C_15_H_25_NO_3_ |  | 37350-58-6 | Sigma Aldrich, Oslo,  Norway | 1.79 | -0.25 | 0.28 |
| Sulfamethoxazole (SMX) | - antibiotic | - C_10_H_11_N_3_O_3_S | **** | 723-46-6 | Sigma Aldrich, Oslo,  Norway | 0.89 | -0.56 | 0.52 |
| Atorvastatin calcium salt trihydrate (ATO) | antilipidemic | C₆₆H₆₈CaF₂N₄O₁₀ |  | - 134523-03-8 | Toronto Research Chemicals, Toronto, Canada | 4.13 | 1.25 | 0.64 |
| Losartan potassium (LOS) | - Anti-hypertensive | - C_22_H_22_ClKN_6_O |  | 124750-99-8 | Sigma Aldrich, Oslo,  Norway | 3.56 | 1.29 | 1.17 |
| Citalopram | - Antidepressant | - C_20_H_21_FN_2_O |  | 59729-33-8 | Sigma Aldrich, Oslo,  Norway | 2.51 | 1.27 | 1.10 |

| **Cpd Name** | **RT** | **Prec Ion** | **Prod Ion1** | **Prod Ion 2** | **Prod Ion 3** | **Frag (V)** | **CE (V) 1** | **CE (V) 2** | **CE (V) 3** | **Polarity** |
| --- | --- | --- | --- | --- | --- | --- | --- | --- | --- | --- |
| Losartan | 5.0 | 423.1 | 377 | 207 | 191.9 | 380 | 13 | 24 | 41 | + |
| Sulfamethoxazole-^13^C_6_ | 6.2 | 260 | 161.1 | 114 | 97.9 | 380 | 13 | 24 | 28 | + |
| Sulfamethoxazole | 6.3 | 254 | 156 | 108 | 92 | 380 | 11 | 22 | 27 | + |
| Carbamazepine-d_10_ | 6.9 | 247.1 | 204 | 202 |  | 380 | 21 | 39 |  | + |
| Carbamazepine | 6.1 | 237 | 193.9 | 178.9 |  | 380 | 18 | 39 |  | + |
| Caffeine ^13^C_3_ | 4.0 | 198.1 | 140.1 | 112.1 |  | 380 | 18 | 25 |  | + |
| Caffeine | 4.1 | 195.1 | 138.1 | 110.1 |  | 380 | 18 | 24 |  | + |
| Warfarin | 6.0 | 307 | 249.9 | 160.9 |  | 380 | 20 | 15 |  | - |
| Diclofenac | 7.9 | 296 | 249.9 | 214.9 | 213.8 | 380 | 9 | 17 | 35 | + |
| Diclofenac-^13^C_6_ | 7.9 | 316 | 272.1 |  |  |  |  |  |  | - |
| Metoprolol | 5.2 | 268.1 | 116.1 | 73.9 |  | 380 | 16 | 21 |  | + |
| Citalopram | 6.4 | 325.2 | 262 | 109 |  | 380 | 15 | 35 |  | + |
| Ibuprofen | 16 | 559.5 | 292 | 250 |  | 380 | 35 | 45 |  | + |
| Acetaminophen | 2.5 | 152.2 | 110 | 65 |  | 380 | 15 | 35 |  | + |
| Atorvastatin | 6.2 | 205 | 161.1 |  |  | 380 | 1 |  |  | - |

**Table S2 MRM Parameters of the selected analytes and their internal standards**

## **S5. Method Validation**

# The method performance characteristics which are listed in Table S3 were determined as follows. Due to the lack of pharmaceuticals free fecal sludge samples, cow manure mixed with bark was used in the method validation. The average percent recovery with relative standard deviation (RSD) was calculated form six replicates samples fortified with a mixture of target compounds and their respective ISTDs at 200 ng g^-1^. Instrument limit of detection (ILOD) and Instrument limit of quantification (LOQ) were determined as the concentration of the pharmaceutical dissolved in 20% CH_3_CN in water that gave a signal/noise ratio (S/N=3) of 3 and 10, respectively. The method detection limit (MDL), was determined as the concentration the pharmaceutical prepared in samples that gave S/N=3. Matrix match and solvent matched calibration curves for targeted analytes were prepared using 10 concentration levels in the rang (0.200- 2000 ng mL^-1^). The recovery percentages of ISTDs (sulfamethoxazole-(phenyl-^13^C_6_), caffeine-^13^C_3_, carbamazepine-D_10_, diclofenac-(acetophenyl ring-^13^C_6_)) were calculated based on their calibration curves over five concentration levels (10, 20, 50, 80, and 100 ng mL^-1^) applying ^2^H_10_-DEET at a concentration of 100 ng mL^-1^ as a recovery standard. In order to determine the influence of matrix on method performance, matrix effect (ME%) was estimated using the equation depicted below, where Ss and Sm are the slope of the solvent matched calibration and matrix matched curves respectively. Positive and negative ME% values indicate signal enhancement and ion suppression by the matrix respectively.

$$ME\%=\left[ \left( \frac{Sm}{Ss} \right)-1 \right]\times100$$

The performance of extraction efficiency of the optimized method was evaluated in terms of Acetaminophen caffeine, carbamazepine, metoprolol, losartan, atorvastatin, warfarin showed satisfactory average recoveries ranged from 38 -124%, and the majority of their RSDs were below 20%; however, a few compounds demonstrated unacceptable recoveries, including diclofenac, ibuprofen, sulfamethoxazole, citalopram. Therefore, the concentration obtained for these compounds are considered semi-quantitative data. It has been widely reported that pharmaceutical compounds analyzed in environmental samples suffer from significant matrix effects resulting in either ionization suppression or enhancement, when analyzed by LC-ESI-MS/MS. In the current study, isotopically labeled internal standards were used to compensate for potential losses during the sample preparation and differences in ionization of the analytes between different samples. All selected compounds experienced significant ionization suppression. However, as matched isotopically labeled ISTDs were not available for each target compound, matrix matched calibration is adopted to account for these matrix effects. The average absolute sample specific recovery % of carbamazepine-D_10_ was found to be 47.0±15%.

## **S6. Removal**

Removal was calculated based on the equation:

Removal % = (C_0_-C)/C_0_

Where C_0_ is the initial concentration of the compound and C is the actual concentration of the compound.

**Table S3 Summary of the method performance characteristics; Instrument limits of detection (LOD) and quantification (LOQ), method detection limit (MDL), and the percent recoveries ± relative standard deviations (RSD).**

| **Compound** | **LOD (ng mL^-1^)** | **LOQ (ng mL^-1^)** | **MDL (ng g^-1^)** | **Matrix Effect** | **(Recovery± RSD, n=6) %** |
| --- | --- | --- | --- | --- | --- |
| Acetaminophen | 0.028 | 0.093 | 0.171 | -91 | 38.4±9 |
| Caffeine | 0.025 | 0.082 | 0.341 | -89 | 62.9±10 |
| Carbamazepine | 0.002 | 0.006 | 0.007 | -95 | 119±8 |
| Diclofenac | 0.306 | 1.020 | 1.020 | -75 | 195±5 |
| Ibuprofen | 10.34 | 34.4 | 13.6 | -82 | - |
| Metoprolol | 0.005 | 0.016 | 0.424 | -81 | 112+9 |
| Sulfamethoxazole | 0.007 | 0.024 | 2.41 | -97 | - |
| Citalopram | 3.48 | 11.6 | 18.7 | -50 | - |
| Losartan | 0.247 | 0.823 | 0.823 | - | 107±46 |
| Atorvastatin | 0.064 | 0.215 | 0.215 | - | 119±37 |
| Warfarin | 0.004 | 0.012 | 0.012 | - | 124±6 |

1. Predicted data is calculated with ACD/Labs Percepta Platform − PhysChem Module, Toronto, CA. ([http://www.chemspider.com/Chemical Structure.18219.html](http://www.chemspider.com/Chemical%20Structure.18219.html)) [↑](#footnote-ref-1)
2. Structures were prepared using ChemDraw Professional (PerkinElmer Informatics, In. version 15.0.9.106, Boston, Massachusetts, USA) [↑](#footnote-ref-2)
